# Systematic profiling of a lipid metabolism‐derived signature guides risk‐stratification and therapeutic strategies in hepatocellular carcinoma

**DOI:** 10.1002/ctm2.1254

**Published:** 2023-05-10

**Authors:** Zhengyi Zhu, Yuyan Chen, Jinkun Xia, Jinglin Wang, Haozhen Ren

**Affiliations:** ^1^ Department of Hepatobiliary Surgery Affiliated Drum Tower Hospital Medical School Nanjing University Nanjing China; ^2^ Nanjing Drum Tower Hospital Clinical College of Nanjing University of Chinese Medicine Nanjing China

## LETTER‐TO‐EDITOR WITH PREVIOUS SUBMISSION NUMBER: CTM2‐2023‐02‐0184

### Dear Editor

Hepatocellular carcinoma (HCC) ranks as the third most lethal cancer with 15% 5‐year survival.^1^ Lipid metabolism dysfunction is a pivotal feature in hepatocarcinogenesis.^2^ Integrating lipid‐related transcriptomics with clinicopathological data is crucial for HCC precision medicine and personalized therapy.^3^ Hitherto, limited research on hepatic lipid metabolism hindered identifying unique HCC subtypes for tailored therapies. Thus, we concentrated efforts on constructing a novel prognostic signature for HCC, capable of predicting optimal adjuvant treatment modalities for particular patients.

**FIGURE 1:**
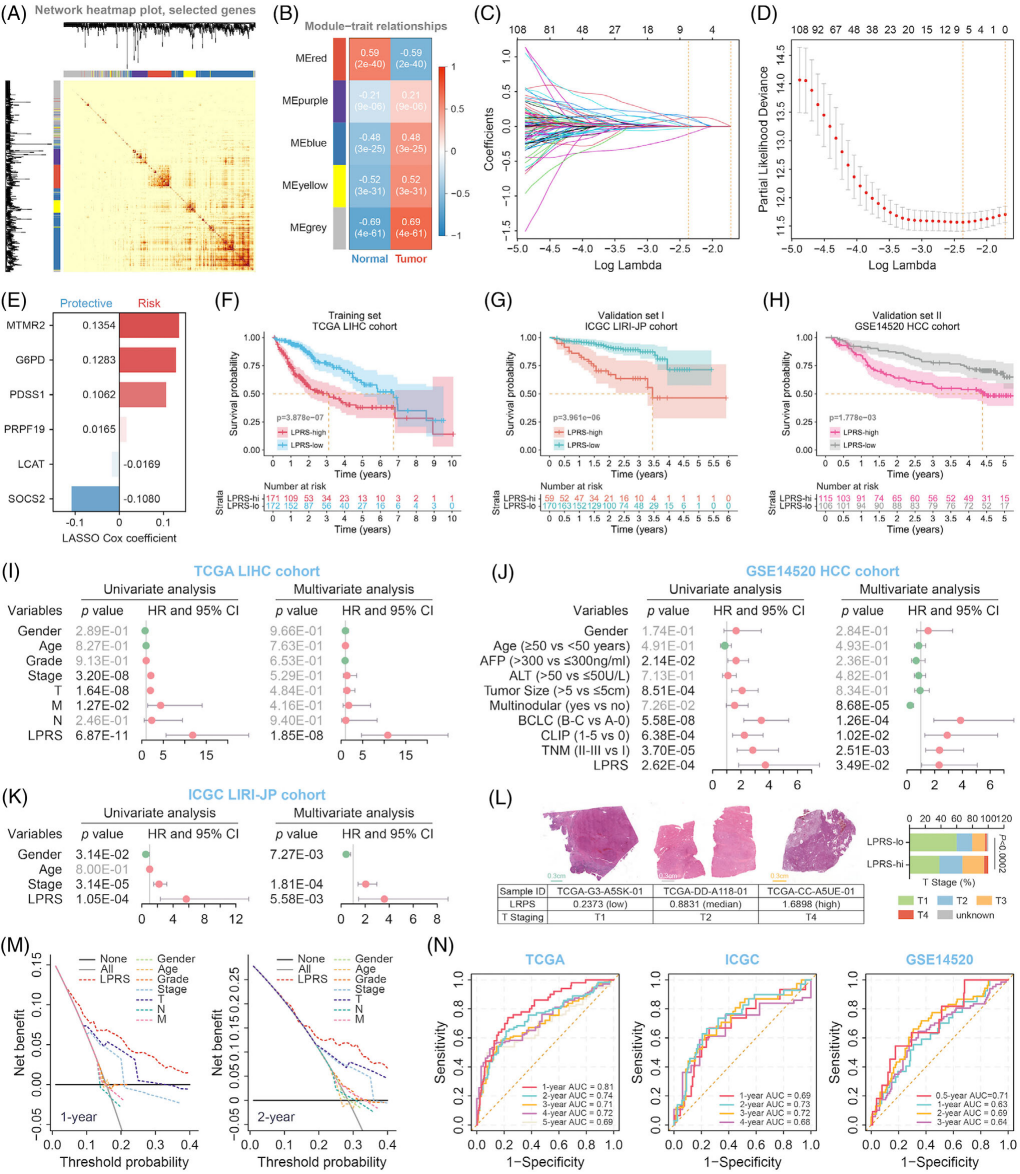
Construction and validation of lipid‐derived prognostic risk score (LPRS) signature. (A) Heatmap of the topological overlap matrix of differentially expressed genes (DEGs) was generated using the WGCNA algorithm. (B) Module‐trait relationships between modules and tumour phenotype. (C) Identification of the most robust prognostic genes by LASSO Cox regression algorithm. (D) Ten‐fold cross‐validation was applied and an optimal λ value of .0938 was selected. (E) Distribution of individual coefficients of the LPRS‐signature. (F) Kaplan–Meier analysis revealed that higher LPRS indicated significantly poorer prognosis in the training cohort, and (G and H) the prognostic value was verified in two independent external validation cohorts. (I) Univariate and multivariate analyses of clinicopathologic factors and LPRS were conducted in the derivation cohort, and (J and K) validated in the validation sets. (L) Representative pathology sections and differences of T staging between two subgroups. (M) Decision curve analyses (DCA) illustrated that LPRS brought more net survival benefits than other parameters. (N) receiver operating characteristic (ROC) analyses of LPRS on OS in all cohorts.

In this study (see Figure S1 for workflow), we retrospectively analyzed the transcriptome data and clinical parameters obtained from The Cancer Genome Atlas (TCGA), ICGC, and GEO portals. We first screened Gene Ontology (GO) annotations with known genes engaged in cellular lipid metabolism (Table S1). The Upset plot illustrated gene list intersection, and three GO terms were integrated to generate the union set that recapitulated lipid‐related genes (Figure S2A). We identified 693 differentially expressed genes (DEGs) of 1467 lipid metabolism‐related genes in TCGA HCC specimens compared to normal tissues (Figure S2B). GO and KEGG pathway enrichment analyses were conducted to dissect the specific biological implications of these genes (Figure S2C,D). We next employed the weighted gene co‐expression network analysis (WGCNA) algorithm to construct a scale‐free co‐expression network and explore DEGs’ correlations with HCC phenotype, and four non‐grey modules were obtained with optimal soft threshold *β* = 7 (Figure 1A,B, Figure S3A). The red, yellow and blue modules were regarded as HCC‐associated modules and network analysis was depicted in Figure S3B. Univariate Cox regression analysis identified 186 lipid‐related tumour‐specific candidate prognostic genes (Figure S3C).

To identify robust prognostic genes, least absolute shrinkage and selection operator (LASSO) regression was employed to build a novel classifier. Ten‐fold cross‐validation was applied to avoid over‐fitting, selecting an optimal λ value of .0938 (Figure 1C,D). A total of six genes were integrated to calculate the lipid‐derived prognostic risk score (LPRS) by the linear combination of gene expressions weighted by corresponding coefficients (Figure 1E). The correlation network was illustrated in Figure S3D. Using the established formula, LPRS‐high and ‐low subgroups were identified by taking the median LPRS as the cut‐off value. Survival analyses demonstrated that patients with higher LPRS experienced worse clinical outcomes (Figure 1F–H). LPRS distribution, survival statuses, clinical features, and gene expression were visualized in Figure S3E–G. Notably, T staging differed considerably among the two subgroups (Figure S3E, Figure 1L). Univariate and multivariate Cox regression analyses verified LPRS as an independent risk factor (Figure 1I–K). The decision curve analyses (DCA) indicated that LPRS conferred more net survival benefits than other indicators (Figure 1M). Meanwhile, the time‐dependent area under ROC curves (AUC) proved that LPRS functioned as a satisfactory prognostic index with high robustness (Figure 1N). To facilitate risk quantification, we constructed a nomogram that integrated the LPRS together with clinical information (Figure S4A). Calibration curves depicted high accuracy in survival probability estimation, and DCA demonstrated the nomogram's superior net benefit to LPRS (Figure S4B,C). Higher nomogram points distinguished worse patient outcomes, and ROC curve analysis showed remarkable discrimination ability (Figure S4D,E).

**FIGURE 2:**
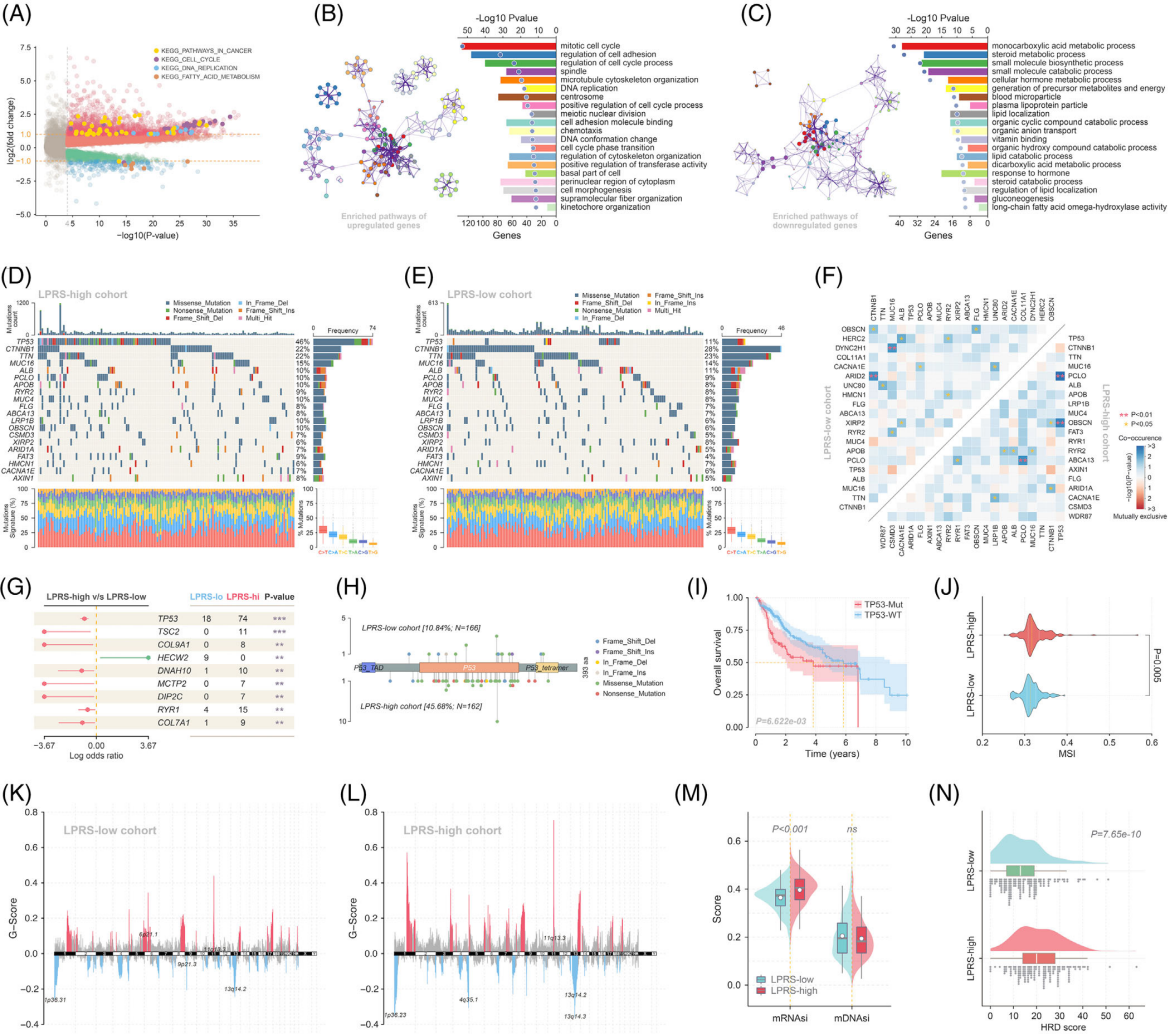
Comparisons of oncogenic features among lipid‐derived prognostic risk score (LPRS)‐stratified subgroups. (A) The volcano plot illustrated differentially expressed genes (DEGs) and representative pathways between LPRS‐defined groups in the TCGA cohort. (B and C) GO enrichment analyses for significantly up‐regulated and down‐regulated genes in LPRS‐high patients compared to the LPRS‐low group. (D and E) Top‐ranked twenty most frequently mutated genes were showcased in LPRS‐defined subtypes. (F) A heatmap of the mutually exclusive or co‐occurring mutations between top frequently mutated genes in subgroups. (G) TP53 was situated in the first position among differently mutated genes between LPRS‐high and ‐low groups. (H) A lollipop plot illustrated the TP53 mutation hotspots in both cohorts. (I) Patients with TP53 mutations experienced worse outcomes than wild‐type patients. (J) MSI was significantly elevated in the LPRS‐high cohort. (K–L) Somatic copy number amplifications and deletions in LPRS‐stratified subgroups. (M) Comparisons of stemness indices and (N) HRD scores between LPRS‐high and ‐low cohorts.

Given the apparent differences in prognoses between LPRS‐stratified subgroups, we subsequently probed the underlying mechanisms. Differential gene expression analysis identified 1637 prominently DEGs (Figure 2A). Metascape conducted GO enrichment analyses, revealing up‐regulated genes in LPRS‐high patients enriched in cancer‐related pathways (e.g., mitotic cycle, adhesion, and DNA replication), while down‐regulated genes were enriched in metabolic pathways involving lipids (Figure 2B,C). Top‐ranked genes with high mutation frequencies were accessed, and TP53 mutations were more frequent in the LPRS‐high cohort (Figure 2D,E), validated by Fisher's exact test (Figure 2G). Co‐occurring TP53, OBSCN, and PCLO mutations were detected in the LPRS‐high cohort (Figure 2F). The lollipop plot showcased TP53 mutations’ frequency and hotspots (Figure 2H), and shorter survival was observed in TP53‐mutated patients (Figure 2I). Furthermore, elevated microsatellite instability (MSI), CNV, stemness indices, and homologous recombination deficiency (HRD) were shown in the LPRS‐high subtype (Figure 2J–N), elucidating other possible carcinogenic mechanisms.

**FIGURE 3:**
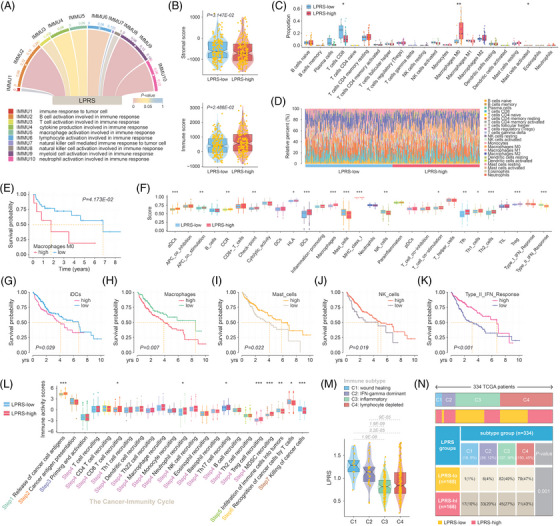
Characteristics of immune cell infiltrates in lipid‐derived prognostic risk score (LPRS)‐stratified subgroups. (A) Correlation coefficients between LPRS and various immune response‐related signatures. (B) Stromal and immune scores calculated by ESTIMATE algorithm. (C and D) The proportions of immune cell subsets in different LPRS subgroups. (E) Kaplan–Meier survival analysis with the proportion of M0 macrophage as the grouping factor. (F) Certain gene signatures were applied to define immune functions between LPRS‐defined subtypes. (G and K) Survival analyses stratified by ssGSEA scores of iDCs, macrophages, mast cells, natural killer (NK) cells or type II interferon (IFN) response. (L) Enrichment scores of steps in the cancer‐immunity cycle between LPRS‐stratified groups. (M) Variations of LPRS among immune subtypes, and (N) distributions of immune subtypes between LPRS‐stratified cohorts.

Intratumoural immune cells are crucial for HCC development and prognosis.^4^ Utilizing GSVA, we correlated LPRS with immune response‐related signatures and determined positive associations with B cells, myeloid cells, and neutrophils (Figure 3A). A larger stromal fraction and more immune infiltration were detected in the LPRS‐low and ‐high subgroups, respectively (Figure 3B). We dissected immune cell subsets in LPRS‐stratified subtypes, discovering that M0 macrophages were more abundant in the LPRS‐high group, while CD8+ T cells were prevalent in the LPRS‐low ones (Figure 3C,D). Similarly, we compared the ssGSEA scores of 29 immune signatures between LPRS‐defined subgroups (Figure 3F). Herein, survival curves were robustly separated by stratification of patients based on immune cell subsets (Figure 3E) and immune signatures (Figure 3G–K). Cancer‐immunity cycle tracks tumour immunity and infiltrates for evaluation.^5^ By comparing cancer‐immunity cycle scores, we recognized that the LPRS‐high patients had up‐regulated antigen release and immune cell trafficking, but down‐regulated immune cell infiltration, cancer cell recognition, and cancer cell killing, partially explaining the worse prognosis (Figure 3L). Based on Thorsson et al.’s study,^6^ immune subtypes were discernible in LPRS‐stratified subgroups (Figure 3 M,N).

**FIGURE 4:**
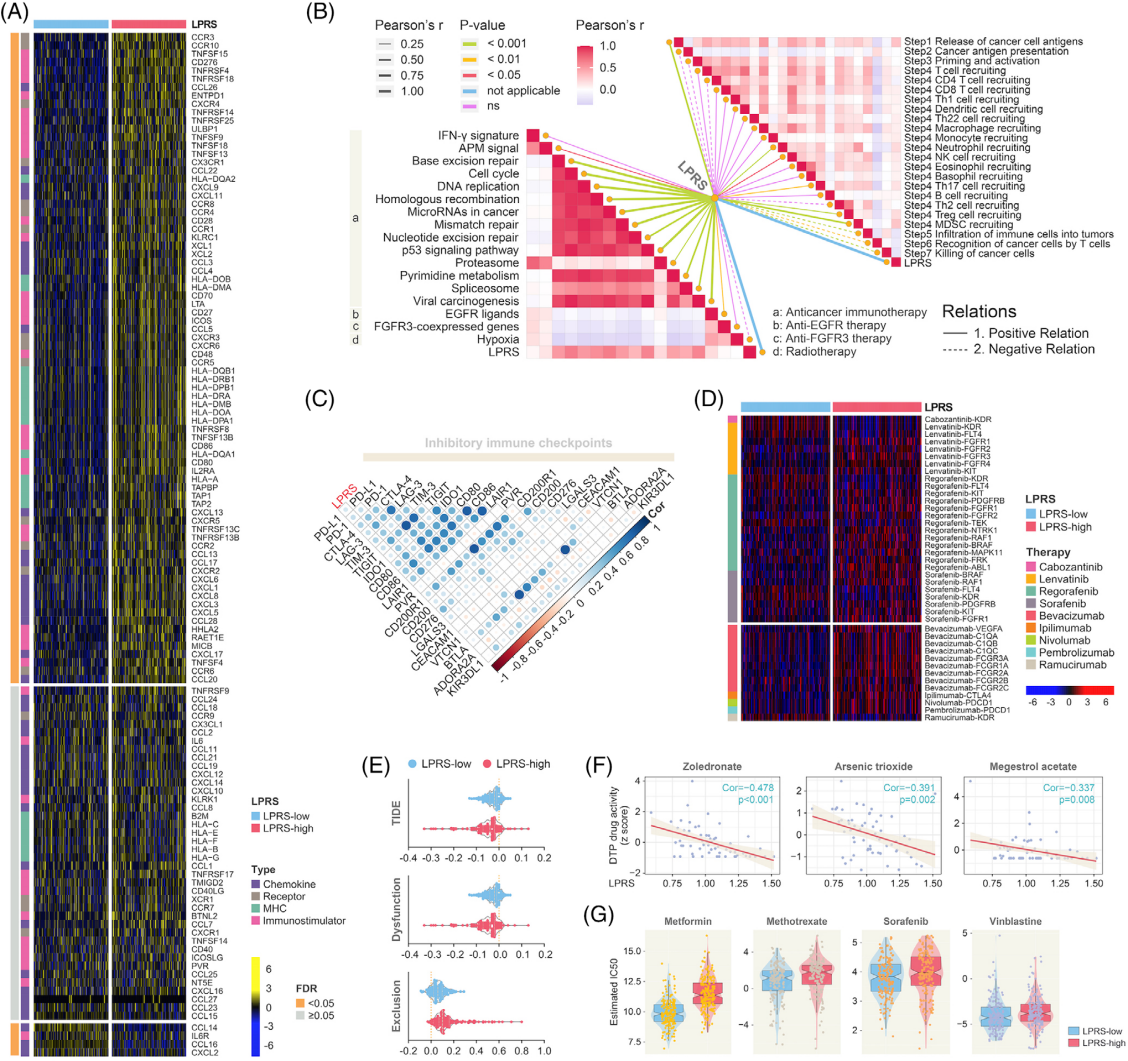
Abilities of lipid‐derived prognostic risk score (LPRS) in predicting therapeutic benefits. (A) Differential expression profiling of immunostimulators between LPRS‐stratified subtypes. (B) Correlations between LPRS and enrichment scores of therapeutic signatures and steps in the cancer‐immunity cycle. (C) Associations between LPRS and inhibitory immune checkpoints. (D) Differential expressions of HCC‐related drug‐target genes obtained from DrugBank in LPRS subgroups. (E) TIDE scores in different LPRS‐stratified cohorts. (F) Correlations between LPRS and activities of specific drugs. (G) Comparisons of estimated IC50 values for several agents between LPRS‐defined subgroups.

Drawing on canonical gene insights,^7^ we examined the expression of immunomodulators and immunostimulators in LPRS‐stratified subtypes (Figure 4A). Positive correlations were observed between LPRS and immune checkpoints CD80, CD86, CD276, and LAIR1, implying immune evasion in the LPRS‐high subgroup (Figure 4C). Therapeutic signatures depicting anti‐cancer immunotherapy were positively correlated with LPRS, highlighting the potential of immunotherapy for LPRS‐high patients (Figure 4B). TIDE algorithm^8^ revealed better immune checkpoint blockade (ICB) response and prolonged survival under anti‐PD1 and anti‐CTLA4 therapies in the LPRS‐high group (Figure 4E), while lenvatinib, regorafenib and sorafenib showed potential drug susceptibilities in these patients (Figure 4D). Moreover, the activities of several adjuvant drugs were negatively correlated with LPRS, while LPRS‐high patients were less vulnerable to certain agents (Figure 4F–G), which indicated heightened drug resistance.

Collectively, compelling evidence demonstrated that LPRS scoring system exhibited a robust value for survival prediction, offered promising capabilities for comprehensive characterization of oncogenic features, and prospectively stratified patients into different subgroups to determine the administration of adjuvant therapies, especially anti‐cancer immunotherapy. Using it to characterize the heterogeneity of HCC may provide valuable clues for treatment decision‐making and eventually facilitate personalized management.

## CONFLICT OF INTEREST STATEMENT

The authors have declared that no competing interest exists.

## FUNDING INFORMATION

The National Natural Science Foundation of China, Grant Numbers: 82100664 and 82270646; The Fundamental Research Funds for the Central Universities, Grant Number: 0214‐14380510; The Nanjing Health Science and Technology Development Project for Distinguished Young Scholars, Grant Number: JQX19002; The Natural Science Foundation of Jiangsu Province, Grant Number: BK20190114; Jiangsu Province Postdoctoral Research Funding Program, Grant Number: 2021K116B; Medical Science and Technology Development Foundation, Nanjing Department of Health, Grant Number: YKK19070; Project of Modern Hospital Management and Development Institute, Nanjing University and Aid project of Nanjing Drum Tower Hospital Health, Education and Research Foundation, Grant Number: NDYG2020047; The Affiliated Drum Tower Hospital, Medical School of Nanjing University, Grant Numbers: 2021‐LCYJ‐PY‐46 and 2022‐LCYJ‐PY‐35; The Chen Xiao‐ping Foundation for the Development of Science and Technology of Hubei Province, China, Grant Numbers: CXPJJH121001‐2021073 and CXPJJH122002‐019

## Supporting information

Supporting Information

Supporting Information

Supporting Information

Supporting Information

Supporting Information
