# Supplementary figures and images for: Systematic profiling of a lipid metabolism‐derived signature guides risk‐stratification and therapeutic strategies in hepatocellular carcinoma
Source: Clin Transl Med. 2023 May 10;13(5):e1254. doi: 10.1002/ctm2.1254 (PMC10172611; doi:10.1002/ctm2.1254)

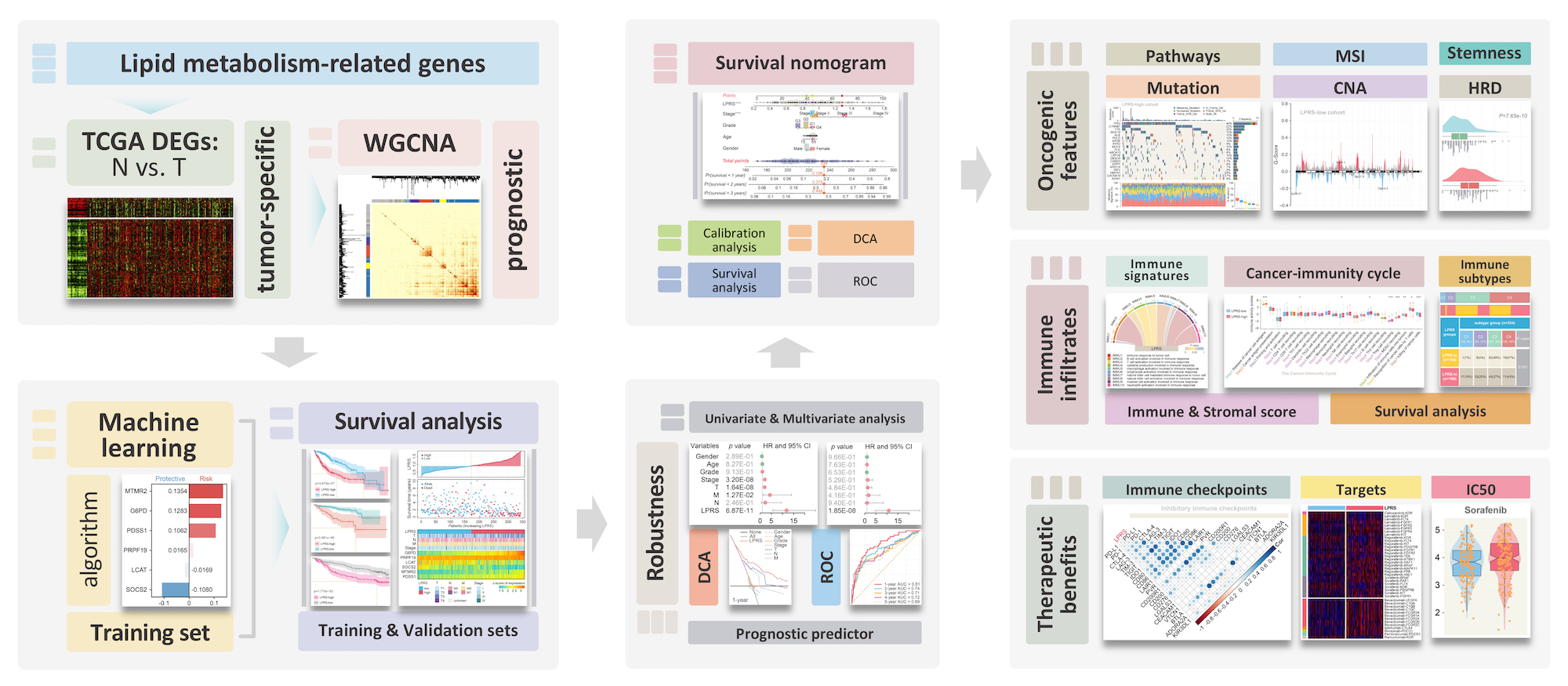

Supplement: Supplementary file 1 — Supporting Information [file CTM2-13-e1254-s003.jpg]

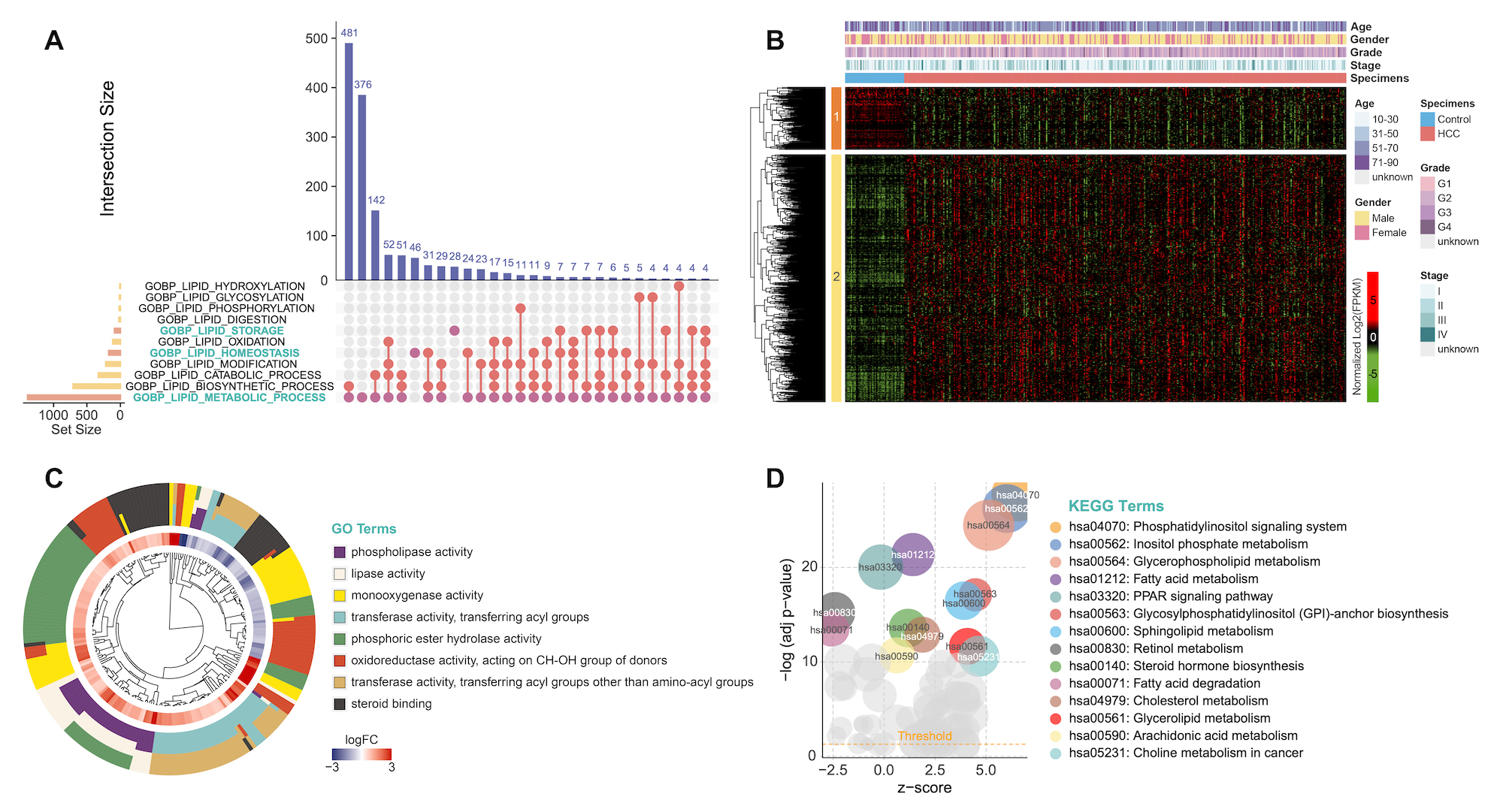

Supplement: Supplementary file 2 — Supporting Information [file CTM2-13-e1254-s005.jpg]

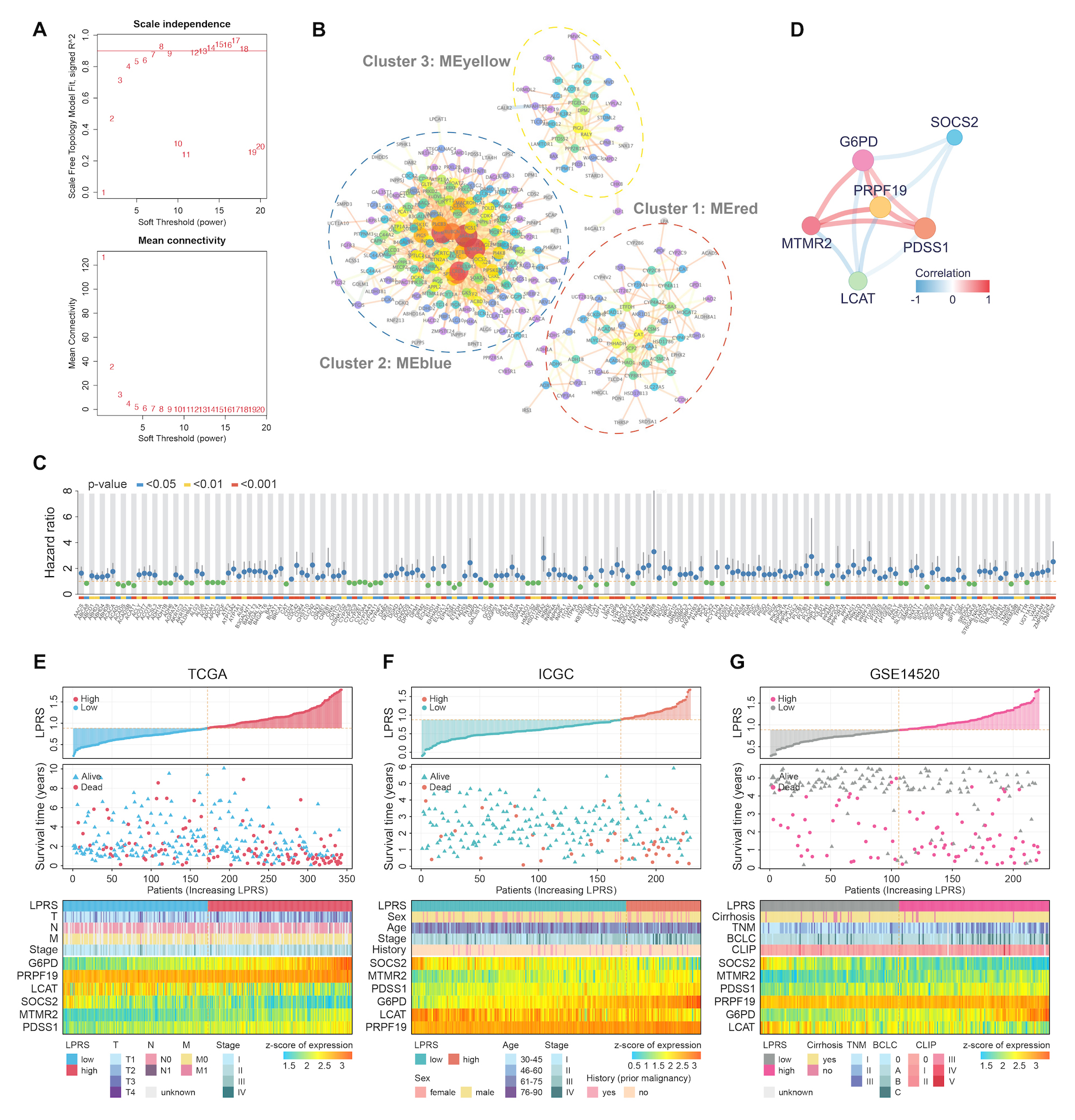

Supplement: Supplementary file 3 — Supporting Information [file CTM2-13-e1254-s001.jpg]

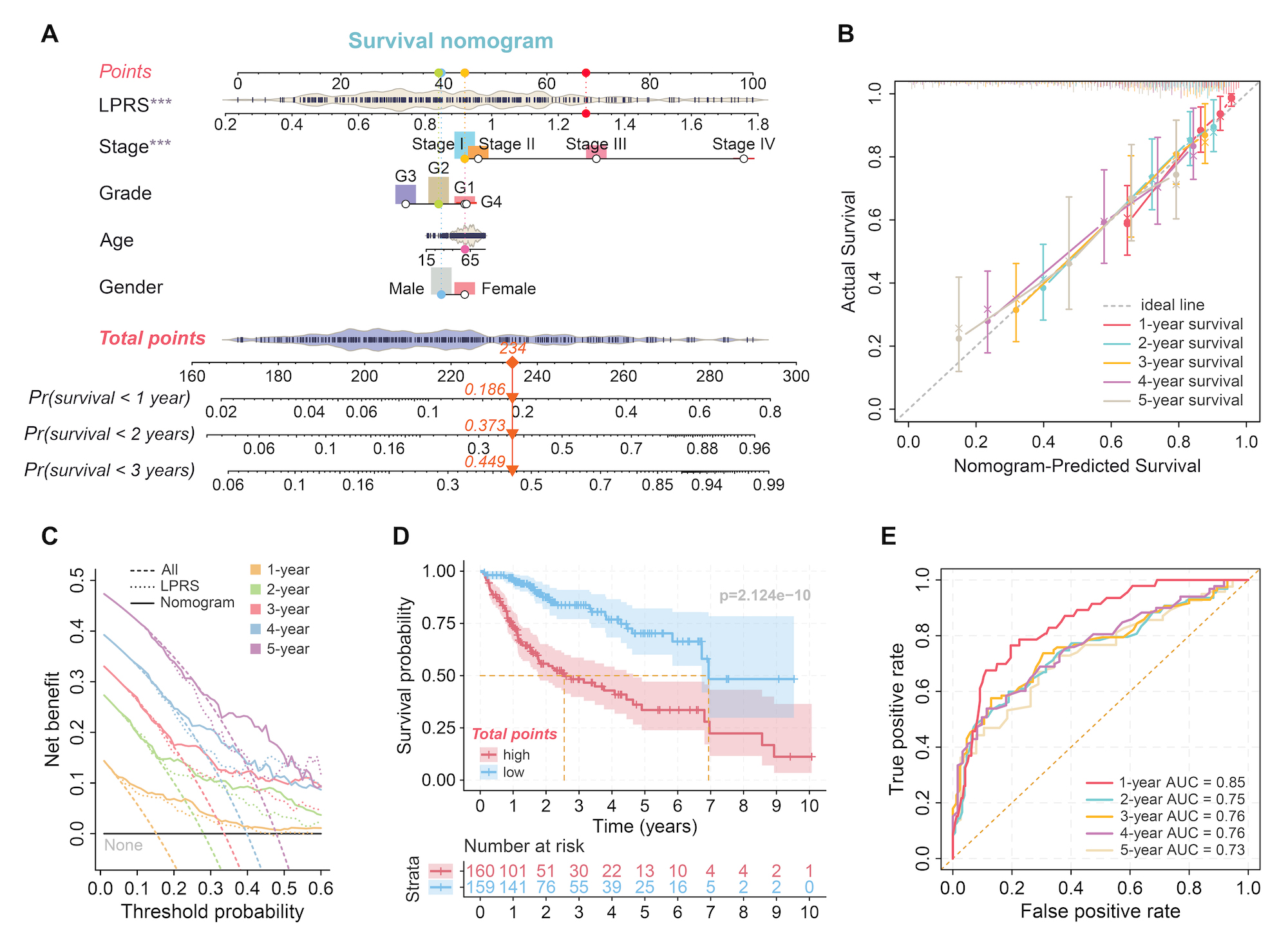

Supplement: Supplementary file 4 — Supporting Information [file CTM2-13-e1254-s004.jpg]
