# Supplementary material for: Systematic profiling of a lipid metabolism‐derived signature guides risk‐stratification and therapeutic strategies in hepatocellular carcinoma
Source: Clin Transl Med. 2023 May 10;13(5):e1254. doi: 10.1002/ctm2.1254 (PMC10172611; doi:10.1002/ctm2.1254)
Supplement: Supplementary file 5 — Supporting Information [file CTM2-13-e1254-s002.docx]

Table S1. Lists of selected lipid-derived GO-BP terms.

| GOBP_LIPID_BIOSYNTHETIC_PROCESS |
| --- |
| > The chemical reactions and pathways resulting in the formation of lipids, compounds soluble in an organic solvent but not, or sparingly, in an aqueous solvent. [GOC:go_curators] |
| GOBP_LIPID_CATABOLIC_PROCESS |
| > The chemical reactions and pathways resulting in the breakdown of lipids, compounds soluble in an organic solvent but not, or sparingly, in an aqueous solvent. [GOC:go_curators] |
| GOBP_LIPID_DIGESTION |
| > The whole of the physical, chemical, and biochemical processes carried out by living organisms to break down ingested lipids into components that may be easily absorbed and directed into metabolism. [GOC:go_curators] |
| GOBP_LIPID_GLYCOSYLATION |
| > Covalent attachment of a glycosyl residue to a lipid molecule. [GOC:mah] |
| GOBP_LIPID_HOMEOSTASIS |
| > Any process involved in the maintenance of an internal steady state of lipid within an organism or cell. [GOC:BHF, GOC:rl] |
| GOBP_LIPID_HYDROXYLATION |
| > The covalent attachment of a hydroxyl group to one or more fatty acids in a lipid. [GOC:hjd, PMID:15658937] |
| GOBP_LIPID_METABOLIC_PROCESS |
| > The chemical reactions and pathways involving lipids, compounds soluble in an organic solvent but not, or sparingly, in an aqueous solvent. Includes fatty acids; neutral fats, other fatty-acid esters, and soaps; long-chain (fatty) alcohols and waxes; sphingoids and other long-chain bases; glycolipids, phospholipids and sphingolipids; and carotenes, polyprenols, sterols, terpenes and other isoprenoids. [GOC:ma] |
| GOBP_LIPID_MODIFICATION |
| > The covalent alteration of one or more fatty acids in a lipid, resulting in a change in the properties of the lipid. [GOC:mah] |
| GOBP_LIPID_OXIDATION |
| > The removal of one or more electrons from a lipid, with or without the concomitant removal of a proton or protons, by reaction with an electron-accepting substance, by addition of oxygen or by removal of hydrogen. [GOC:BHF, GOC:mah] |
| GOBP_LIPID_PHOSPHORYLATION |
| > The process of introducing one or more phosphate groups into a lipid, any member of a group of substances soluble in lipid solvents but only sparingly soluble in aqueous solvents. [GOC:bf, ISBN:0198506732] |
| GOBP_LIPID_STORAGE |
| > The accumulation and maintenance in cells or tissues of lipids, compounds soluble in organic solvents but insoluble or sparingly soluble in aqueous solvents. Lipid reserves can be accumulated during early developmental stages for mobilization and utilization at later stages of development. [GOC:dph, GOC:mah, GOC:tb, PMID:11102830] |
